# Investigation of NF-κB-94ins/del ATTG and CARD8 (rs2043211) Gene Polymorphism in Acute Lymphoblastic Leukemia

**DOI:** 10.3389/fendo.2019.00501

**Published:** 2019-08-02

**Authors:** Chen Zhang, Fengjiao Han, Jie Yu, Xiang Hu, Mingqiang Hua, Chaoqin Zhong, Ruiqing Wang, Xueyun Zhao, Yufeng Shi, Chunyan Ji, Daoxin Ma

**Affiliations:** ^1^Department of Hematology, Qilu Hospital of Shandong University, Jinan, China; ^2^Institute for Financial Studies and School of Mathematics, Shandong University, Jinan, China; ^3^Shandong Provincial Key Laboratory of Immunohematology, Qilu Hospital of Shandong University, Jinan, China

**Keywords:** NLRP3, polymorphism, NF-κB, CARD8, acute lymphoblastic leukemia

## Abstract

NLRP3 inflammasome has been widely implicated in the development and progression of various hematological diseases. However, how NLRP3 inflammasome contributes to the pathogenesis and clinical features of acute lymphoblastic leukemia (ALL) is still unknown. Here, in ALL patients' bone marrow, we investigated the single-nucleotide polymorphisms (SNPs) and expression of NLRP3 inflammasome related genes, NF-κB, NLRP3, IL-1β, IL-18, Caspase-1, and ASC. A total of 308 ALL patients and 300 healthy participants were included in this study. D allele and DD genotype under codominant model of NF-κB-94ins/del ATTG were showed as a protective factor in susceptibility of ALL. As for CARD8 (rs2043211), AT/TT genotype under dominant model and TT genotype under codominant model greatly increased the ALL susceptibility. We further studied the relationship between NLRP3 inflammasome genetic polymorphisms and clinical relevance. The results showed that DD genotype of NF-κB-94 ins/del ATTG and AT/TT genotype of CARD8 (rs2043211) contributed to lower WBC count and T-cell immunophenotype, respectively. Moreover, we also found that AT and TT genotypes of CARD8 (rs2043211), GT and TT genotypes of IL-1β (rs16944), and TT genotype of IL-18 (rs1946518) were associated with higher mRNA expression of NLRP3 inflammasome related genes and secretion of downstream cytokines. In conclusion, NF-κB-94 ins/del ATTG and CARD8 (rs2043211) genotypes might serve as novel biomarkers and potential targets for ALL.

## Introduction

Acute lymphoblastic leukemia (ALL) is a clonal disorder driven by accumulation of progenitor cells in the peripheral blood and bone marrow (1, 2). Although adult ALL accounts for only 20% of leukemia, it is more common in childhood acute leukemia (80% of cases). Nowadays, through traditional chemotherapy, the cure rate of ALL can only reach about 20–40% in adults compared to 80% in children (3, 4). Adult patients present higher risk features at diagnosis, more likely to come up with chemotherapy resistance and higher relapse rate after complete remission (CR) (5). Multiple studies reported that the genetic variants of some metabolism and inflammation related genes contribute to ALL pathogenesis and prognosis (6, 7).

Inflammasomes are large cytosolic multiprotein complexes that assemble in response to infection or stress-associated stimuli and lead to the activation of caspase-1-mediated innate immune responses (8). The NOD-like receptor family was the first family of sensor proteins discovered to form inflammasomes. Among the inflammasomes, the NLRP3 inflammasome has been the most thoroughly studied. NLRP3 inflammasome is composed of NLRP3, the adaptor protein ASC (apoptosis-associated speck-like protein containing a CARD) and caspase-1 (9). NLRP3 inflammasome can be activated by exogenous pathogen invasion or *in vivo* cell damage and death, and then lead to the interaction of the PYD domain in NLRP3 and ASC proteins. Assembled inflammasome triggers the cleavage of dormant procaspase-1 into its active form, which turns the inactive proinflammatory cytokines pro-IL-1β and pro-IL-18 into mature and biologically active IL-1β and IL-18, respectively (10, 11). Caspase recruitment domain-containing protein (CARD) 8 interacts with cleaved caspase-1 and then triggers downstream inflammatory response through nuclear factor (NF)-κB pathway (12). NLRP3 inflammasome could modulate cellular levels of the glucocorticoid receptor and affect glucocorticoid resistance of ALL patients (13). However, it is still unknown whether and how NLRP3 inflammasome contributes to the pathogenesis and clinical features of ALL.

Nowadays, the advanced high-throughput techniques have boosted the discovery of genetic variations including copy number variants (CNVs), indels (deletions or insertions), structural variants and single nucleotide polymorphisms (SNPs) (14). SNPs of NLRP3 greatly influence pathogenic challenges and lead to disease outcome, such as autoimmune diseases (15), cardiovascular diseases (16), and malignant tumors (17). The SNP of CARD8 rs2043211 polymorphism has been confirmed to be associated with cardiovascular diseases according to a meta-analysis (18). Accumulating evidences have revealed that NF-κB-94 ins/del ATTG polymorphism is associated with cancer risk such as gastric cancer, hepatocellular carcinoma, and lung cancer (19–21). The polymorphism rs1946518 of NLRP3 effector molecule IL-18 has been shown to influence periodontitis (22) and hepatocellular carcinoma (23). The SNP rs16944 of the other NLRP3 effector molecule IL-1β has been reported contributing to cervical carcinoma (24) and gastritis risk (25). Thus, polymorphism of NLRP3 inflammasome related genes showed strong relevance with malignant tumors, but the regulatory role of the SNP of NLRP3 inflammasome related genes in the ALL bone marrow microenvironment has not been reported until now.

Therefore, to determine the susceptibility and clinical significance of the NLRP3 inflammasome in ALL, we examined the SNPs of four NLRP3 inflammasome related genes, including NF-κB-94ins/del ATTG, CARD8 (rs2043211), IL-18 (rs1946518), and IL-1β (rs16944). We are looking forward to provide evidences for clinical management of ALL.

## Materials and Methods

### Study Subjects and Sample Collection

A total of 308 ALL patients and 300 sex- and age-matched healthy controls were included in this study. The diagnosis of enrolled ALL was based on NCCN Guidelines for acute lymphoblastic leukemia (ALL) (26). The demographic and clinical characteristics of these subjects were shown in Table 1. The protocol was approved by the Medical Ethics Committee of Qilu Hospital of Shandong University. Informed consents were obtained from the participants.

**Table 1:** The demographic and clinical characteristics of ALL patients and controls.

	**ALL patients**	**Controls**
No.	308	300
Age, mean ± SD	38.76 ± 17.02	40.06 ± 12.98
Gender (M/F)	184/124	167/133
WBC count (<30^*^10^9^/L / >30^*^10^9^/L)	240/68	NA
Hb (<60 g/L/ > 60 g/L)	250/58	NA
Cr (<115 g/L/ > 115 g/L)	298/10	NA
Spleen (Splenomegaly/Non-Splenomegaly)	248/60	NA
AST (<125 g/L/>125 g/L)	291/17	NA
Fasting blood-glucose (normal/abnormal)	242/66	NA
Immunophenotype (T/B)	66/242	NA
Ph(Ph+/Ph–)	235/73	NA

For determination of polymorphisms, we collected anti-coagulated bone marrow or peripheral blood from ALL patients or peripheral blood from healthy controls. For testing the association of NLRP3 molecules mRNA expression or plasma levels with the genetic polymorphisms, we used bone marrow samples from newly-diagnosed ALL patients. Bone marrow or peripheral blood samples were centrifuged at 430 g for 5 min, and the plasma supernatants were frozen at −80°C for the determination of cytokines by ELISA. The mononuclear cells were isolated by Ficoll-Hypaque density gradient centrifugation, and re-suspended in sterile PBS and centrifuged again for 5 min at 430 g to remove remaining Ficoll solution. The mononuclear cells were stored −20°C for DNA and RNA extraction.

### DNA Extraction and Genotyping

DNA was extracted using TIANamp Genomic DNA Kit (Tiangen, Beijing, China) according to the supplier's recommendations. The concentration and purity of DNA were accessed at 260/280 absorbance using NanoDrop spectrophotometer. The genotype of CARD8 (rs2043211), IL-18 (rs1946518), or IL-1β (rs16944) was performed using TaqMan® allelic discrimination assay (Cat# 4351379; Thermo Fisher Scientific, USA) in accordance with the manufacturer's instruction. Followed the standard protocol, 3 μl TaqMan Universal PCR Master Mix (2×), 0.15 μl pre-designed primers and probes (40×), 1 μl (100 ng/μl) of DNA and 1.85 μl ddH_2_O make up the 6 μl volume reaction system. The NF-κB-94 ins/del ATTG polymorphism was examined by using the forward primer: 5′-CCG TGC TGC CTG CGT T-3′, reverse primer: 5′-GCT GGA GCC GGT AGG GAA-3′, and probe 1: 5′-VIC- ACC ATT GAT TGG GCC -MGB-3′ and probe 2: 5′-FAM- CGA CCA TTG GGC C -MGB-3′. A total 10 μl volume reaction system was composed of 5 μl TaqMan Universal PCR Master Mix (2×), 0.8 μl (10 μM) forward primer, 0.8 μl (10 μM) reverse primer, 0.2 μl (10 μM) probe 1, 0.2 μl (10 μM) probe 2, 2.0 μl ddH_2_O and 1 μl (100 ng/μl) of DNA. ABI7500 Real-Time PCR system (Applied Biosystems, Foster City, CA, USA) was applied for TaqMan SNP genotyping assay, conditions as followed: 50°C for 2 min, 95°C for 10 min, and then 40 cycles of amplification (92°C denaturation for 15 s, 62°C annealing/extension for 60 s). ABI7500 software v 1.3 and TaqMan Genotyper software were used to analyze the genotyping.

### Real-Time Quantitative PCR Detection for NF-κB, NLRP3, IL-1β, IL-18, Caspase-1, and ASC

We determined the mRNA expression of NF-κB, NLRP3, IL-1β, IL-18, Caspase-1, and ASC by real-time quantitative PCR. RNA was extracted using TRIzol reagent (Invitrogen Life Technologies, Carlsbad, CA). A total of 1 μg RNA was used for reverse transcription of total 10 μl volume by applying the PrimeScript RT reagent Kit Perfect Real Time (Takara Bio, Japan). The quantitative PCR was performed on the LightCycler 480II real-time PCR system (Roche, Switzerland) according to standard protocol. The primers were shown in Table 2. The quantitative PCR is a 10 μl volume, comprised of 5 μl of 2× SYBR Green Real-time PCR Master Mix, 1 μl of cDNA, 0.8 μl of the forward and reverse primers, and 3.2 μl ddH_2_O. The results were expressed relative to the number of GAPDH transcripts used as an internal control.

**Table 2:** The primer sequences of real-time quantitative PCR.

**Gene**	**Forward (5^**′**^-3^**′**^)**	**Reverse (5^**′**^-3^**′**^)**
NF-κB	TCC AGA CCA ACA ACA ACC CC	GAT CTT GAG CTC GGC AGT GT
NLRP3	CAG ACT TCT GTG TGT GGG ACT GA	TCC TGA CAA CAT GCT GAT GTG A
IL-1β	GCC CTA AAC AGA TGA AGT GCT C	GAA CCA GCA TCT TCC TCA G
IL-18	GCT TGA ATC TAA ATT ATC AGT C	GAA GAT TCA AAT TGC ATC TTA T
Caspase-1	AAA TCT CAC TGC TTC GGA CAT G	GGA ACT TGC TGT CAG AGG TCT T
ASC	TGG ATG CTC TGT ACG GGA AG	CCA GGC TGG TGT GAA ACT GAA
GAPDH	GCT CTC TGC TCC TCC TGT TC	GTT GAC TCC GAC CTT CAC CT

### ELISA for IL-18 and IL-1β

The levels of IL-18 and IL-1β in plasma were determined with a quantitative sandwich enzyme-linked immunosorbent assay (ELISA) in accordance with the manufacturer's recommendations (lower detection limits were 62.5 pg/ml for IL-18 and 3.9 pg/ml for IL-1β; IL-18 ELISA kit was bought from Abcam and IL-1β ELISA kit was bought from R&D systems, USA).

### Statistical Analysis

The *p* value of the Hardy–Weinberg equilibrium (HWE) was calculated using the calculator available at the Helmholtz Center Munich website. In addition to allelic frequencies, we analyzed genotypic frequencies under three genetic models, specifically, the codominant, dominant, and recessive models. Association between the SNPs and ALL susceptibility was calculated by a chi-squared (χ2) test or a Fisher's exact test. Univariate logistic regression analysis was used to analyze *p* values and odds ratios (ORs) with a corresponding 95% confidence interval (95% CI). A two-tailed *p* < 0.05 was considered statistically significant. All statistical analysis was performed using SPSS 22.0 software (SPSS Inc., Chicago, IL, USA).

## Results

### The Polymorphisms of NF-κB-94 ins/del ATTG and CARD8 (rs2043211) Contribute to the Susceptibility of All

We examined SNPs of NLRP3 inflammasome related genes: NF-κB (-94 ins/del ATTG), CARD8 (rs2043211), IL-18 (rs1946518), and IL-1β (rs16944) in 308 ALL patients and 300 healthy controls. The distribution of AT/TT genotype under dominant and TT genotype under codominant models for rs2043211 in CARD8 were found significantly different between ALL patients and controls. In addition, both the D allele in allelic frequencies and DD genotype under the codominant model of NF-κB−94 ins/del ATTG were significantly associated with the susceptibility to ALL (*p* < 0.05; Table 3).

**Table 3:** The association between selected SNPs and susceptibility of ALL.

**SNP**	**Model/Allele**	**Genotype/Allele**	**Controls**		**ALL patients**		***P* value**
			**Count**	**%**	**Count**	**%**	
NF-kB 94ins/delATTG	Allele	W	339	56.5	383	62.2	
		D	261	43.5	233	37.8	0.047
	Codominant	WW	98	32.7	119	38.6	
		WD	143	47.7	145	47.1	
		DD	59	19.6	44	14.3	0.027
CARD 8rs2043211	Dominant	AA	93	31	62	20.1	
		AT+TT	207	69	246	79.9	0.002
	Codominant	AA	93	31	62	20.1	
		AT	134	44.7	172	55.8	
		TT	73	24.3	74	24	0.004

Univariate logistic regression analysis was used for analyzing NF-κB and CARD8. For 94 ins/del ATTG in NF-κB, the frequency of D allele was lower in ALL patients compared to controls (37.8 vs. 43.5%), and it was significantly associated with ALL susceptibility (OR = 0.790, 95% CI 0.628–0.994, *p* = 0.044). Furthermore, the frequency of the DD genotype of NF-κB (14.3 vs. 19.6%) was obviously lower in ALL rather than homozygote insertion or wildtype (WW) genotype under codominant model, which hinted a significant association with ALL susceptibility (OR = 0.641, 95% CI 0.383–0.946, *p* = 0.043). For CARD8 under dominant model, the AT/TT genotype was significantly higher in ALL patients compared to controls (79.9 vs. 69.0%), and was associated with ALL susceptibility (OR = 1.783, 95% CI 1.230–2.583, *p* = 0.002). As for codominant model, both AT and TT genotypes of CARD8 (55.8 vs. 44.7%; 24.0 vs. 24.3%, respectively) were much higher in ALL compared to controls, and it significantly contributed to ALL susceptibility rather than AA genotype (OR = 1.925, 95% CI 1.300–2.583, *p* = 0.001; OR = 1.521, 95% CI 0.964–2.399, *p* = 0.072, respectively; Table 4). Interestingly, the distribution of CARD8 rs2043211 and NF-κB-94 ins/del ATTG was found different between ALL patients and controls. NF-κB polymorphism demonstrated a protective effect, while AT/TT genotype of CARD8 (rs2043211) greatly increased the ALL susceptibility.

**Table 4:** NF-κB-94 ins/del ATTG and CARD8 (rs2043211) contribute to susceptibility of ALL.

**SNP**	**Model/Allele**	**Genotype/Allele**	**Controls**		**ALL patients**		**OR (95%CI)**	***P* value**
			**Count**	**%**	**Count**	**%**		
NF-kB 94ins/delATTG	Allele	W	339	56.5	383	62.2	1.000	
		D	261	43.5	233	37.8	0.790 (0.628–0.994)	0.044
	Codominant	WW	98	32.7	119	38.6	1.000	
		WD	143	47.7	145	47.1	0.835 (0.568–1.189)	0.317
		DD	59	19.6	44	14.3	0.614 (0.383–0.986)	0.043
CARD8rs2043211	Dominant	AA	93	31	62	20.1	1.000	
		AT+TT	207	69	246	79.9	1.783 (1.230–2.583)	0.002
	Codominant	AA	93	31	62	20.1	1.000	
		AT	134	44.7	172	55.8	1.925 (1.300–2.852)	0.001
		TT	73	24.3	74	24	1.521 (0.964–2.399)	0.072

### DD Genotype of NF-κB-94 ins/del ATTG and AT/TT Genotype of CARD8 (rs2043211) Contribute to Lower WBC Count and T-Cell Immunophenotype, Respectively

According to NCCN Guidelines for ALL, several prognostic indicators are listed to indicate the prognosis. Our study analyzed the associations between these prognostic indicators and four SNPs of NLRP3 inflammasome genes, aimed to figure out the correlation between NLRP3 genetic polymorphisms and prognosis of ALL.

We found that NF-κB-94 ins/del ATTG was significantly associated with the lower white blood cells (WBC) count, which is supposed to be a poor prognostic indicator as long as WBC count over 30^*^10^9^ /L. After univariate logistic regression analysis, compared with WD/WW genotype of NF-κB under recessive model, DD genotype significantly associated with a lower WBC count (20.0 vs. 5.9%; OR = 0.250, 95% CI 0.087–0.721, *p* = 0.010). Under codominant model, DD genotype also associated with a lower WBC count compared to WW genotype (16.7 vs. 5.9%; OR = 0.325, 95% CI 0.403–0.998, *p* = 0.048). NF-κB-94 ins/del ATTG under both recessive and codominant model, patients with DD genotype tend to have a lower WBC count (Table 5).

**Table 5:** The polymorphisms of NF-κB-94 ins/del ATTG and CARD8 (rs2043211) were associated with prognosis characteristics of ALL.

**SNP**	**Model/Allele**	**Genotype/Allele**	**Clinical characteristic**				**OR (CI95%)**	***P* value**
			**Count**	**%**	**Count**	**%**		
NF-kB 94ins/del ATTG	Recessive		WBC Count <30		>30			
		WW+WD	192	80	64	94.1	1.000	
		DD	48	20	4	5.9	0.250 (0.087–0.721)	0.010
	Codominant		WBC Count <30		>30			
		WW	91	37.9	28	41.2	1.000	
		WD	109	45.4	36	52.9	1.073 (0.609–1.892)	0.807
		DD	40	16.7	4	5.9	0.325 (0.107–0.998)	0.048
CARD8 rs2043211	Dominant		IPT (B-cell)		IPT (T-cell)			
		AA	55	22.7	7	10.6	1.000	
		AT+TT	187	77.3	59	89.4	2.479 (1.071–5.738)	0.034
	Codominant		IPT (B-cell)		IPT (T-cell)			
		AA	55	22.7	7	10.6	1.000	
		AT	127	52.5	45	68.2	2.748(1.182–6.559)	0.019
		TT	60	24.8	14	21.2	1.833(0.689–4.876)	0.225

Furthermore, CARD8 (rs2043211) was also found to be correlated with immunophenotype of ALL, which primary divided into T-cell immunophenotype and B-cell immunophenotype. According to NCCN Guideline for ALL, immunophenotype is a strong prognostic indicator. Compared with AA genotype under dominant model, AT/TT genotype was associated with T-cell immunophenotype (77.3 vs. 89.4%; OR = 2.479, 95% CI 1.071–5.738, *p* = 0.034). Under codominant model, AT genotype was also associated with T-cell immunophenotype compared to AA genotype (52.5 vs. 68.2%; OR = 2.748, 95% CI 1.182–6.559, *p* = 0.019; Table 5). Thus, DD genotype of NF-κB-94 ins/del ATTG was showed as a protective factor, while AT/TT genotype of CARD8 (rs2043211) associated with a poor prognosis.

### NF-κB-94 ins/del ATTG, IL-18 (rs1946518) and IL-1β (rs16944) Contribute to Clinical Characteristics of All Patients

The NLRP3 inflammasome genetic polymorphisms are also correlated with other clinical indicators of diagnosis, treatment approaches, and complication management. For NF-κB-94 ins/del ATTG, univariate logistic regression analysis revealed that DD genotype (*p* = 0.046) was statistically associated with hemoglobin (Hb) compared to WW/WD genotype under recessive model. Under codominant model, DD genotype (*p* = 0.019) was significantly associated with splenomegaly in contrast to WW genotype. As for the frequency of allele, compared with the−94insATTG (W) allele, the minor allele homozygotes were also associated with splenomegaly (*p* = 0.045; Table 6).

**Table 6:** The association between selected SNPs and clinical characteristics of ALL patients.

**SNP**	**Model/Allele**	**Genotype/Allele**	**Clinical characteristics**				**OR (CI95%)**	***P* value**
			**Count**	**%**	**Count**	**%**		
NF-kB	Recessive		Hb <60		>60			
94ins/delATTG		WW+WD	43	74.1	213	85.2	1.000	
		DD	15	25.9	37	14.8	0.498 (0.251–0.986)	0.046
			Splenomegaly		Non-Splenomegaly			
	Codominant	WW	99	39.9	20	33.3	1.000	
		WD	120	48.4	25	41.7	1.031 (0.541–1.966)	0.926
		DD	29	11.7	15	25	2.560 (1.165–5.625)	0.019
	Allele		Splenomegaly		Non-Splenomegaly			
		W	318	64.1	65	54.2	1.000	
		D	178	35.9	55	45.8	1.512 (1.010–2.262)	0.045
IL-18 rs1946518	Recessive		Splenomegaly		Non-Splenomegaly			
		GG+GT	194	78.2	38	63.3	1.000	
		TT	54	21.8	22	36.7	2.080 (1.135–3.811)	0.018
			AST <125		>125			
		GG+GT	223	76.6	9	52.9	1.000	
		TT	68	23.4	8	47.1	2.915 (1.083–7.848)	0.034
	Codominant		Glu <6.1		>6.1			
		GG	59	24.4	22	33.3	1.000	
		GT	121	50	34	51.5	0.754 (0.405–1.401)	0.371
		TT	62	25.6	10	15.2	0.433 (0.189–0.990)	0.047
	Allele		Glu <6.1		>6.1			
		G	239	49.4	78	59.1	1.000	
		T	245	50.6	54	40.9	0.675 (0.457–0.998)	0.049
IL-1β rs16944	Recessive		Cr <115		>115			
		GG+GT	219	73.5	4	40	1.000	
		TT	79	26.5	6	60	4.158 (1.143–15.122)	0.031
	Allele		Cr <115		>115			
		A	302	50.7	5	25	1.000	
		G	294	49.3	15	75	3.082 (1.106–8.587)	0.031

Under recessive model, TT genotype of IL-18 (rs1946518) was statistically correlated with both splenomegaly and AST concentration in serum compared with GG/GT genotype (*p* = 0.018, *p* = 0.034, respectively). In addition, TT genotype was also found to be related to the fasting blood-glucose under codominant model compared to GG genotype (*p* = 0.047). As for the frequency of allele, compared with the G allele, the T allele homozygote was also associated with the fasting blood-glucose (*p* = 0.049; Table 6).

Furthermore, the polymorphism in IL-1β rs16944 was supposed to have connection with creatinine concentration. Under recessive model, TT genotype (*p* = 0.031) was significantly associated with creatinine concentration by the contrast to GG/GT genotype. As for the frequency of allele, the G allele was also associated with creatinine concentration (*p* = 0.031; Table 6).

### Association Between the Polymorphisms and the Expression of NLRP3 Inflammasome Related Genes

The pathogenesis and development of ALL involves a dysregulated immune microenvironment, which contains the production of multiple immunological effector proteins and the activation of the downstream pathways. To further comprehend the relationship between NLRP3 inflammasome related gene polymorphisms and its effective proteins, we use RT-PCR to examine NLRP3 inflammasome related gene expression at mRNA level, and ELISA to detect the secretion of effective proteins.

**Figure 1:**
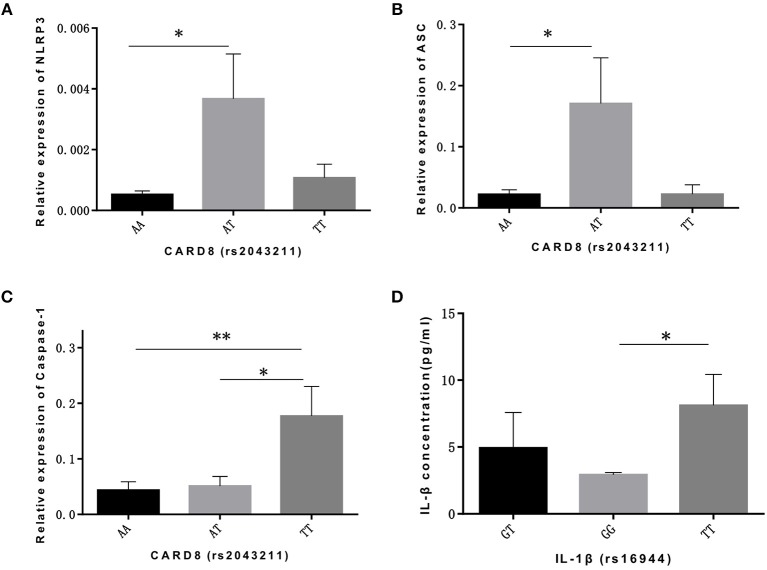
CARD8 polymorphisms (rs2043211) was associated with the mRNA expression ofNLRP3 inflammasome related genes and its effective cytokines in ALL patients bone marrow microenvironment. AT genotype was correlated with higher mRNA expression ofNLRP3 **(A)** and ASC **(B)** compared to AA genotype, while TT genotype was correlated with higher caspase-1 **(C)** mRNA expression capared with AA and AT genotypes. TT genotype also contributed to the higher IL-18 concentration **(D)** in ALL patients bone marrow microenvironment. ^*^p < 0.05; ^**^p < 0.01.

As for CARD8 (rs2043211), AT genotype showed a significant association with the higher mRNA expression levels of NLRP3 and ASC compared to AA genotype (*p* = 0.017, *p* = 0.024, respectively; Figures 1A,B). We also found that TT genotype was correlated with higher caspase-1 mRNA level when compared to both AA and AT genotype (*p* = 0.009, *p* = 0.039, respectively; Figure 1C). Moreover, TT genotype in CARD8 polymorphism was also found to be associated with IL-18 concentration in ALL bone marrow microenvironment compared to AA genotype (Figure 1D).

**Figure 2:**
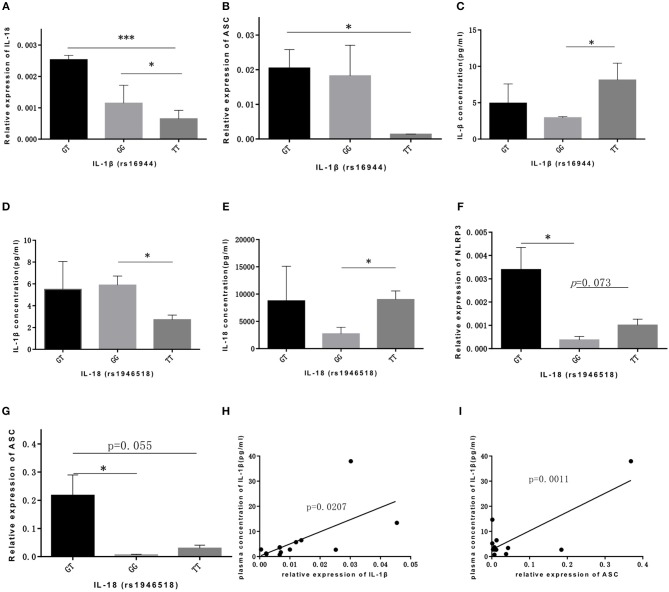
IL-lP (rsl6944) and IL-18 (rsl946518) were associated with higher mRNA expression ofNLRP3 related genes and secreted cytokines. GT and GG genotypes of IL-lP (rsl6944) were associated with mRNA expression levels ofiL-18 **(A)** and ASC **(B)** compared to TT genotype. IL-l p concentration **(C)** was higher in ALL patients bone marrow with TT genotype. IL-l p concentration **(D)** or IL-18 concentration **(E)** were higher in GG or TT genotype of IL-18 (rs1946518) polymorphisms. NLRP3 **(F)** and ASC **(G)** mRNA expression levels were significant high in GT genotype ofiL-18 (rsl946518) polymorphisms. IL-lP concentration correlated with mRNA expression of IL-l p **(H)** and ASC **(I)**. ^*^p < 0.05; ^***^p < 0.001.

As for IL-1β (rs16944) polymorphism, GT genotype was significantly associated with IL-18 expression compared with GG and TT genotype (*p* = 0.021, *p* = 0.000, respectively; Figure 2A), and was also associated with ASC expression compared with TT genotype (*p* = 0.045; Figure 2B). Our data showed that TT genotype was correlated with IL-1β concentration (*p* = 0.044; Figure 2C). With the similar trend, TT genotype in IL-18 (rs1946518) polymorphism was also associated with both IL-1β and IL-18 concentration compared to GG genotype (*p* = 0.029, *p* = 0.022, respectively; Figures 2D,E). However, GT genotype in IL-18 polymorphism was related to a higher level of NLRP3 and ASC (*p* = 0.034, *p* = 0.048, respectively; Figures 2F,G). Besides, we also found significant correlation between concentration of IL-1β in bone marrow microenvironment and mRNA expression level of IL-1β and ASC (*p* = 0.020, *p* = 0.001, respectively; Figures 2H,I). Thus, AT and TT genotype of CARD8 (rs2043211), GT and TT genotype of IL-1β (rs16944) and TT genotype of IL-18 (rs1946518) were associated with higher mRNA expression of NLRP3 related genes and secreted cytokines.

## Discussion

ALL is a malignant blood disease with origins from somatic genetic alterations in bone marrow microenvironment (27). Though traditional chemotherapy has made a great progress in the last decade, ALL patients still have a poor prognosis. Compared with 90% 5-year overall survival in children, it is merely 30–40% in adult ALL. NLRP3 inflammasome systematically exists in human organism, and its activation has been identified as initiation of numerous diseases, such as immune diseases (28), cardiovascular diseases (29), gut homeostasis (30), and even malignant cancers (31). In hematological diseases, genetic polymorphisms of the NLRP3 have been reported to contribute to the pathogenesis of multiple myeloma (32) and lymphoma (33). But what is the correlation between NLRP3 inflammasome and ALL is still unknown. Therefore, our study mainly focused on the relationship between NLRP3 inflammasome and ALL in order to depict the internal communication between them.

We found that the DD genotype and D allele in NF-κB-94 ins/del ATTG was associated with a decreased risk of ALL, and had been considered as a protective factor. With the similar trend, under both recessive mode and codominant model, DD genotype exhibits as a protective factor on WBC count and Hb concentration. NF-κB-94 ins/del ATTG is considered as a protective factor for ALL patients. NF-κB is not only the downstream of NLRP3 inflammasome, but also is involved in many immune and inflammatory responses. In the resting state, NF-κB was found in cytoplasm, combined with several regulatory proteins, which inhibit its function (34, 35). After stimulating factors activate inflammatory responses, it moves into nucleus and binds to specific DNA sequences to modulate apoptosis and proliferation related genes (36). As NF-κB-94 ins/del ATTG polymorphism was determined as a protective factor in our study, this gene polymorphism may result in low affinity to nucleus; however, further studies need to be performed to illuminate its mechanisms.

On the contrary, CARD8 (rs2043211), another polymorphism we focused on, seems to increase the risk for ALL according to our study. It should be noted that TT genotype under codominant model increased the susceptibility of ALL, showed strong positive correlation with caspase-1 mRNA expression and the concentration of IL-18 in bone marrow microenvironment. CARD8 polymorphism was supposed to positively enhanced the NLRP3 inflammasome activation and the susceptibility of ALL.

Since we are aimed to provide new horizons for clinical management of ALL patients, we focused on several clinical indicators that may be related to prognosis. Though there were still many indicators we studied not listed in NCCN Guideline of prognostic indicators, they are significantly related to complications or the survival quality of ALL patients. According to our study, polymorphisms of IL-18 and IL-1β should be significantly associated with blood routine index, biochemical criterion.

In conclusion, our investigation found that NLRP3 inflammasome-related SNPs, especially NF-κB-94ins/del ATTG and CARD8 (rs2043211) genotype might serve as a novel biomarker and potential targets for ALL. Furthermore, IL-1β (rs16944) and IL-18 (rs1946518) polymorphism are able to predict the prognosis and guide the treatment of ALL. Further research with a larger sample size and in-depth mechanism study is required to validate the clinical potential of IL-1β (rs16944) and IL-18 (rs1946518) polymorphism.

## Ethics Statement

This study was carried out in accordance with the recommendations of CIOMS Guideline. The protocol was approved by the Laboratory Animal Ethic Committee of Qilu Hospital of Shandong University.

## Author Contributions

DM and CJ conceived and designed the experiments. CZha performed the experiments. RW and FH analyzed the data. YS, CZho, and MH contributed reagents, materials, and analysis tools. XH and XZ wrote the paper. All authors read the manuscript critically and approved the submitted version.

### Conflict of Interest Statement

The authors declare that the research was conducted in the absence of any commercial or financial relationships that could be construed as a potential conflict of interest.
